# Sea foam contains hemoglycin from cosmic dust†

**DOI:** 10.1039/d4ra06881e

**Published:** 2024-11-20

**Authors:** Julie E. M. McGeoch, Malcolm W. McGeoch

**Affiliations:** a High Energy Physics DIV, Smithsonian Astrophysical Observatory Center for Astrophysics|Harvard & Smithsonian 60 Garden Str, MS 70 Cambridge MA 02138 USA julie.mcgeoch@cfa.harvard.edu; b PLEX Corporation 275 Martine Str, Suite 100 Fall River MA 02723 USA

## Abstract

In-falling cosmic dust has left evidence of meteoritic polymer amide in stromatolites, both fossil and modern. In search of evidence for continued present day in-fall, sea foam was collected from two beaches in Rhode Island and subjected to Folch extraction to concentrate amphiphilic components in a chloroform water–methanol interphase layer. Hemoglycin polymer amide molecules previously characterized by MALDI mass spectrometry in meteorites and stromatolites were identified in sea foam either directly, or *via* their fragmentation patterns. Residual isotope enrichment pointed to an extra-terrestrial origin. The unique resiliency of sea foam may be due to the formation of extended hemoglycin lattices that stabilize its closed-cell structure and its lightness can potentially be explained by photolytic hydrogen production.

## Abstract 2

Sea foam is subjected to Folch extraction and mass spectrometry to isolate hemoglycin, an iron-terminated anti-parallel polymer of glycine first found in meteorites. The polymer has elevated isotope levels indicative of an origin in cosmic dust.

## Graphical abstract

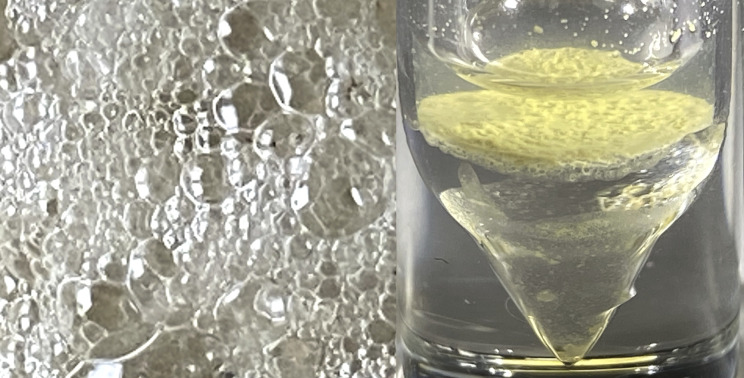

## Introduction

Sea foam, a closed-cell foam that accumulates on shorelines world-wide was investigated to determine whether it contained hemoglycin, a polymer of glycine and iron present in dust arriving from space. Over 5200 metric tons of cosmic dust arrives on the surface of the Earth annually.^1^ The polymer has been extensively characterized from in-fall sources such as carbonaceous meteorites^2–7^ and in fossil and present-day stromatolites.^8^ The polymer may already have been detected by astronomers at wavelengths ranging from UV and visible to the infrared^9^ in molecular clouds and protoplanetary discs. Sea foam is relatively stable and its collection soon after formation, at a shoreline, was pursued as a potential source of the in-fall polymer. Added support for such a polymer sample collection mechanism came from the existence of a surface micro-layer^10^ that may be 50 microns thick carrying accumulated organic molecules and serving as a floating interface between the ocean and the troposphere. Sea foam's appearance on a remote Rhode Island shoreline was noted by the authors to mostly be confined to late autumn into early winter, and this could be related to the axial tilt of the Earth.^11^ A physical resemblance between sea foam structure and hemoglycin vesicles was an additional reason to analyze this foam source. The method whereby hemoglycin has been extracted from meteorite and stromatolite sources *via* a tested solvation system,^2^ in which hemoglycin polymers accumulate at a solvent inter-phase, was applied here to its detection in sea foam.

Sea foam is referenced in a poem by Hesiod 730-700BC and therefore known to exist by the Greeks even if they gave it a mythological context: Θεoγoνíα, Theogonía.^12^ A thorough scientific report on sea foam goes back to Plateau in 1873 (ref. 13) who described the statics of liquids subjected to molecular forces only. The topological rules outlined by Plateau on foam were covered by Hill and Eastoe in 2017 in a review of the stabilization and destabilization of aqueous foams.^14^ The Hill review covers both biological sea foam and man-made foams. The chemical make-up of sea foam from a wide variety of sites in Europe was studied by Mecozzi and Pietroletti,^15^ who applied UV-visible absorption, FTIR and FTNIR spectroscopy to build an extensive data base. FTIR data was particularly revealing as it allowed identification of aliphatic chains, esters and fatty acids, silicates, aromatics, and of particular interest in the present work, the amide I and amide II bands of protein in the region of 1500–1700 cm^−1^. The high protein content of sea foam near kelp beds has been presented by Velimirov^16,17^ and beyond protein its wider chemical diversity is reviewed by Schilling and Zessner.^10^

We hypothesized that the underlying structure of sea foam that only appears in late autumn to early winter in the USA NorthEast could depend upon hemoglycin arriving at the ocean surface *via* a seasonally increased in-fall from space at that time, implying that time-dependent biological components could in many cases be secondary additions to the original structural material of the foam. Bacterial content of sea foam is secondary.^18^ For a recent view of the organic matter in sea foam, NOAA (National Oceanic and Atmospheric Administration) has an update from June 2024.^19^

## Results

**Fig. 1:**
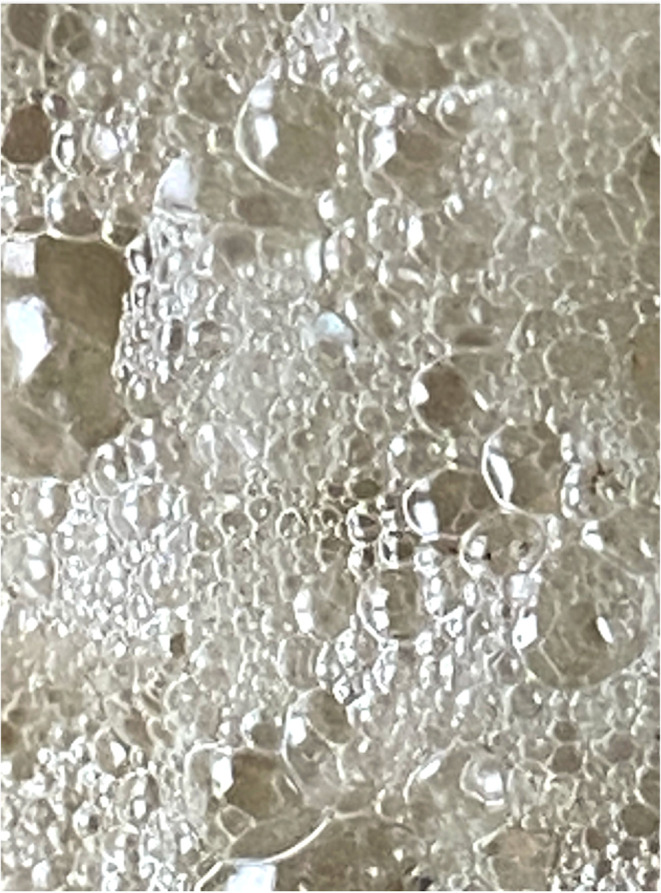
Sea foam sample SF21, showing its faceted structure. Collected at the time point of the 2024 Perseid in-fall^20^ and after a series of RI storms from the remnant of Hurricane Debby.

**Fig. 2:**
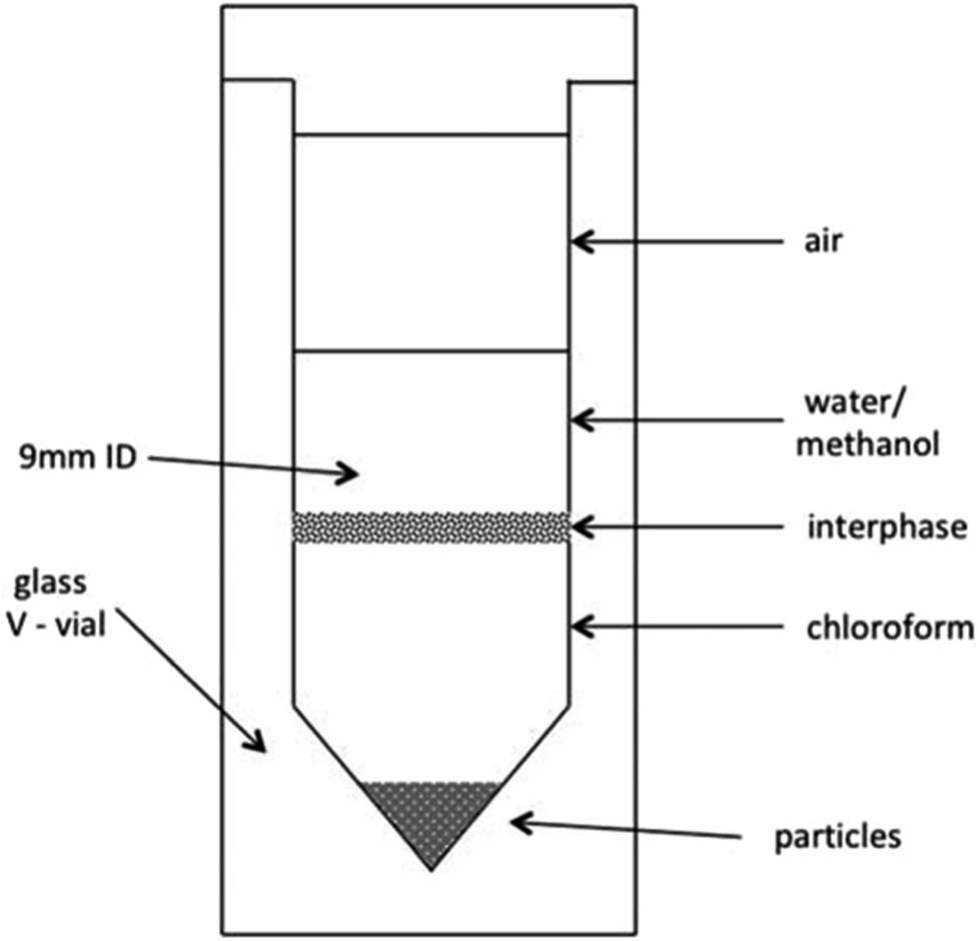
Folch extraction glass V-vial. 3D structures are at the interphase between water/methanol and chloroform. Particles sink in chloroform. From Julie E. M. McGeoch and Malcolm W. McGeoch, (2021) Structural Organization of Space Polymers, *Physics of Fluids*, **33**, 6, June 29th https://aip.scitation.org/doi/10.1063/5.0054860.

**Fig. 3:**
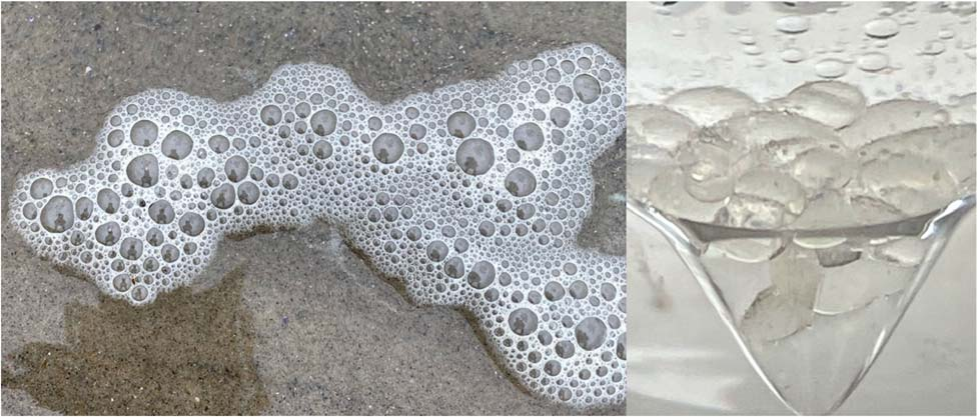
Clean floating sea foam (Sample SF3) was collected directly from the shoreline of the ocean in RI on 2nd December 2023 (left). The wind was from the West. The foam was scooped in a glass bottle, transported to the laboratory, aspirated directly into chloroform in a V-vial and the methanol and water added to give a Folch 2-phase system of chloroform : methanol : water (3.3 : 2 : 1). On vortexing, clear vesicles formed at the Folch interphase (right). The vesicles were stable at room temperature for 1–2 weeks. The intact vesicles were analyzed by MALDI mass spectrometry producing from this sample only the 1657–1663 *m*/*z* sequence.

**Fig. 4:**
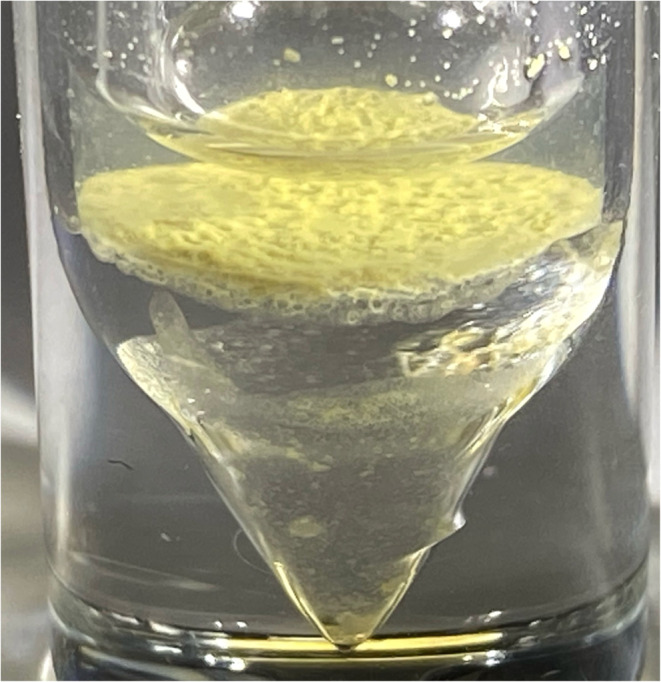
Folch extraction of Sea foam (Sample SF9). Glass V-vial with yellow interphase Folch layer, chloroform below, water–methanol above, air at top.

**Fig. 5:**
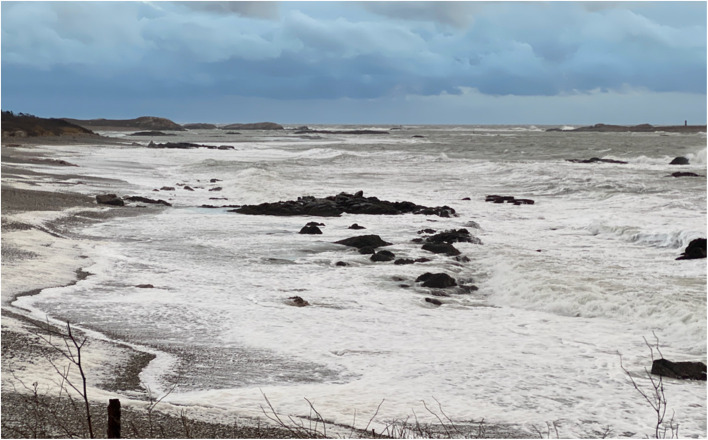
Sea foam, sample SF17, at Lloyds Beach location January 2024. This sea foam stretched more than 100 m out to sea. This location on this day produced foam that was not clean and contained visible sand and plant matter from the turbulent mixing of the sea by the wind and waves.

21 samples of sea foam were collected from two Rhode Island (RI) beaches between September 23rd 2023 and February 27th 2024 (see Methods for the GPS locations and Results Table 1 for a sample list). An outlying sample, sample SF21, was collected in mid-August 2024 after a series of RI storms from the remnant of Hurricane Debby (Table 1) and this coincided with the yearly Perseid in-fall.^20^ The typical faceted appearance of the individual foam cells, with 120° angles, is shown for sample SF21 (Fig. 1). All samples in the 2-phase Folch extraction (Fig. 2 and Methods) produced a typical physical form consistent with the presence of the space polymer hemoglobin, namely stable vesicles^3^ that formed at the interphase layer (Fig. 3). Matrix-assisted laser desorption of ions (MALDI) mass spectrometry was performed on 4 samples with sample SF9 giving the strongest molecular signals from a dense interphase layer (Fig. 4). The presence of high salt from the ocean hampered MALDI analysis in many samples because metal ions can cause the matrix compounds to form complexes,^21^ reducing matrix availability for the protonation of sample molecules. MALDI mass spectrometry is however the best method to obtain information on unknown molecular samples because the separation of molecules and molecule fragments is a single step process avoiding loss of material and the introduction of artifacts associated with other multiple-step mass spectrometry methods. Extensive foam (sample SF17) on a shoreline is shown in Fig. 5 where it extends far out to sea. Due to massive turbulence this sample was a yellow/brown color from sand and plant debris in the foam.

**Table:** Sea foam samples

Sea foam sample	Date of collection	Stable vesicles at Folch interphase	MALDI mass spectrometry
SF1	23.10.24	Yes	
SF2	23.11.21	Yes	
CONTROL sand collected near to sample 2 location	23.11.26	NO	
SF3	23.12.2	Yes	Yes on vesicles at interphase
			Hemoglycin detected
SF4	23.12.10	Yes	
SF5	23.12.11	Yes	
SF6		Yes	
SF7	23.12.19	Yes	
SF7	23.12.20	Yes	
SF7	23.12.21	Yes	
SF8	23.12.23	SF introduced to just chloroform – vesicle bounding sea water above chloroform	Yes on dry crystal after chloroform evaporated after 22 days. Hard crystal not solubilized by matrix mix
SF9	23.12.23	Yes	Yes on interphase samples
			Hemoglycin detected
SF10	23.12.27	Yes	
SF11	24.1.3	Yes	
SF12 sand present	24.1.6	Yes & denser vesicles in chloroform phase	
SF13 sand present	24.1.7	Yes & denser vesicles in chloroform phase	
SF14 sand present	24.1.7	Yes & denser vesicles in chloroform phase	
SF15 sand present sample divided into 2: (1) heated CONTROL. (2) Unheated test. Then both Folch extracted	24.1.11	No vesicles in sample heated in crucible for 10 minutes 500 °C	
		Yes vesicles in test	
SF16	24.1.11	Yellow object on vial wall	Yes but only matrix peaks
	24.1.30		
SF17	24.1.14	Yes	
SF18	24.1.31	Yes	
SF19	24.2.26	Yes	
SF20	24.2.27	Yes	
SF21	24.8.9&10	Yes	Storm from the south coincided with the yearly Perseid in-fall

Two controls were performed. At the exact point of the shoreline of sample 2 collection wet sand was collected 5 days later and subjected to Folch extraction but did not produce vesicles at the interphase. The 2nd control was sample SF15 which was divided into two parts and one half heated in a crucible to 500 °C for 10 minutes. Both parts were Folch extracted with vesicles appearing at the interphase only in the unheated sample. A cylindrical yellow object adhered to the vial wall in sample SF16 which was analyzed by MALDI however hemoglycin was not present in sample SF16 but only matrix peaks were seen. The yellow object was considered to be plant contamination based on the fact it had a regular repeating structure within its length. Samples SF12-15 had some sand present – all produced vesicles at the interphase plus some in the chloroform phase. Because hemoglycin tends to accumulate matter, fine sand particles made the vesicles too heavy to reside at the interphase layer.

### Mass spectrometry

**Fig. 6:**
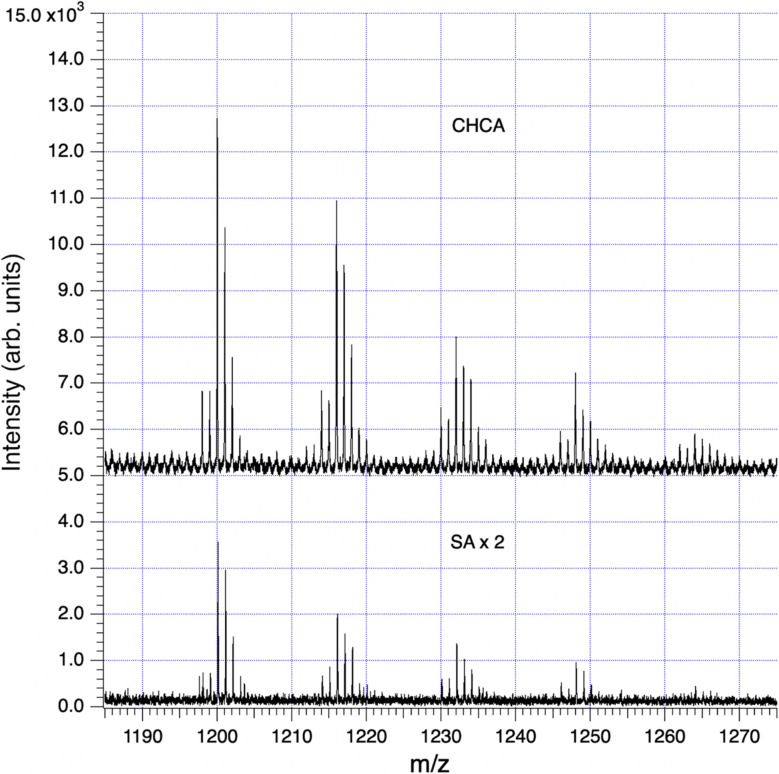
Series of hydroxylations in an 18-residue sea foam analog of the 22-residue hemoglycin core molecule, data in Table 1. The upper (CHCA) trace has been vertically offset for clarity. The lower (SA) trace is shown ×2 for easier comparison.

**Fig. 7:**
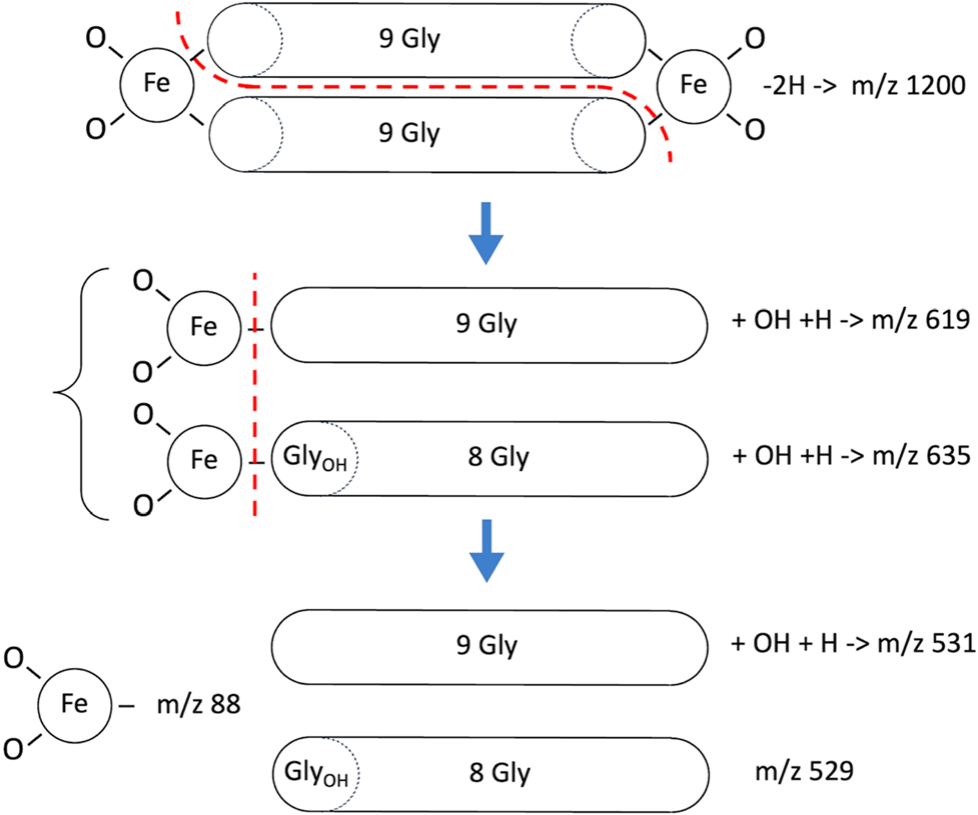
Fragmentation modes of the 1200 *m*/*z* series per Table 2. The single Gly_OH_ residue in *m*/*z* 635 can be situated either adjacent to Fe (shown above), or distally.

Our principal method of molecular analysis is MALDI mass spectrometry, through which intact hemoglycin, its adducts, and its typical fragmentation patterns have been determined in prior work.^2,3,8^ In sample SF9, the analysis with highest signal-to-noise (S/N) ratio, we followed two principal fragmentation patterns that originated respectively from entities at *m*/*z* 1200 and *m*/*z* 1662. The *m*/*z* 1200 peak was the first of a series (Fig. 6) rising in mass increments of 16, that represented successive degrees of oxidation of glycine residues to hydroxyglycine.^3,6^ In work reporting the chiral 480 nm absorption of hemoglycin ^6^ it was determined that a hydroxyglycine residue had to have its C-terminus adjacent to an iron atom in order to create the observed 480 nm absorption. It was therefore assumed that any hydroxylation was present only in the four locations adjacent to terminal iron atoms, as shown for example in Fig. 7. We discuss the 1200 and 1662 *m*/*z* entities in turn.

#### The “1200” *m*/*z* series

In MALDI mass spectrometry there is typically seen a group of primary molecules that are extracted intact from the laser-heated matrix, providing an upper bound to the observed *m*/*z* values, and this group may be accompanied by lower *m*/*z* fragment peaks that help confirm its identity. The matrices, prepared as described in Methods, were CHCA (α-cyano-4-hydroxycinnamic acid) and SA (sinapinic acid) and each matrix was used in duplicate, *e.g.* runs labelled SA-1 and SA-2.

With sample SF9, runs CHCA-1, CHCA-2, SA-1 and SA-2 there was a series of peaks beginning at *m*/*z* 1200 (Fig. 6) that provided an excellent case study of the MALDI fragmentation of hemoglycin. The series was seen with both matrices, CHCA (mass 189) and SA (mass 224), ruling out the possibility that it could relate to matrix clusters. The amplitude of the series decreased steadily as *m*/*z* rose through 1200, 1216, 1232, 1248 and 1264 suggesting a molecular type that could carry increasing levels of oxygenation. In our prior work with hemoglycin^3,8^ we had determined that typically four glycine residues out of 22 were oxidized at the alpha carbon to hydroxyglycine, which has residue mass 73. The “1200” oxygenation series was suggestive of this, and a further aspect of the traces, their (−1) and (−2) isotopologue levels indicated that Fe was present at a level of two or more Fe atoms. The signal-to-noise (S/N) level of the 1200 series itself was poor, and not adequate to determine Fe content by the method described.^3^ However, many of the fragments originating from 1200 *m*/*z* had higher S/N and were analyzed below to obtain Fe content. A trial molecule was written down in Fig. 7 based upon hemoglycin although having 18 residues as opposed to 22 in the usual antiparallel strands closed out by Fe atoms.^3,6^ The context of this length variation will be discussed below.

Analysis of the data in all four of the mass spectra relating to this sample (SF9) produced the consistent set of fragments listed in Table 2. These pointed to a break-up mode into two equal fragments of *m*/*z* in the region of 600, *via* separation along a line of hydrogen bonds between the antiparallel beta strands, plus breakage of two peptide-Fe bonds, shown by the dashed red line in Fig. 7. Subsequent fragmentation occurred *via* the vertical red dashed line in that figure. The expected fragments are all observed, including fragments containing hydroxyglycine and the FeO_2_ fragment that was identified by the exact ^54^Fe (−2) isotope peak intensity at *m*/*z* 86. The “600” *m*/*z* series fragments of Table 2*via* isotope spectrum analysis, Section S1,† contain a single Fe atom, confirming the disposition of Fe atoms, one at each end of the 1200 species. In relation to the primary mode of splitting of hemoglycin, the same hydrogen bond un-zipping mode separating the anti-parallel polyglycine strands was found to be dominant in the splitting of the 22-residue core unit into two fragments at *m*/*z* 730.^3^

**Table:** Within each member of the 1200 *m*/*z* progression the sum of peak counts in a complex comprising mono-isotopic peak and satellites is similarly distributed with either CHCA or SA as the matrix. The principal fragments in the *m*/*z* 600 range depend to a degree on the choice of matrix

*m*/*z*	Molecule	CHCA_1	CHCA_2	SA_1	SA_2
1200	18Gly(FeO_2_)_2_ − 2H	17 700	2900	4700	3300
1216	17GlyGly_OH_(FeO_2_)_2_ − 2H	14 500	2300	2600	2400
1232	16Gly2Gly_OH_(FeO_2_)_2_ − 2H	9800	1000	1600	1300
1248	15Gly3Gly_OH_(FeO_2_)_2_ − 2H	4800	0	900	0
1264	14Gly4Gly_OH_(FeO_2_)_2_ − 2H	1400	0	0	0

	**Fragment**				
600	9Gly(FeO_2_) − H	0	0	5500	3600
619	9Gly(FeO_2_) + OH + H	35 100	13 300	4000	3000
635	8GlyGly_OH_(FeO_2_) + OH + H	30 300	8100	0	1100
88	FeO_2_	74 000	197 000	5200	3400
529	8GlyGly_OH_	31 200	7400	2000	1500
531	9Gly + OH + H	52 000	16 400	4000	3400

#### The “1662” *m*/*z* series

**Fig. 8:**
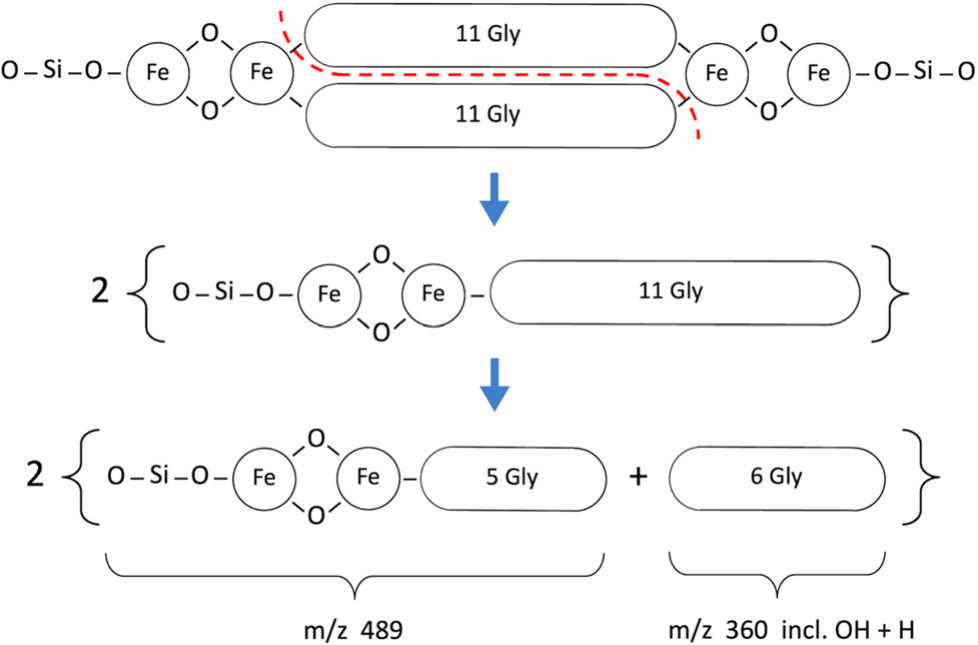
Fragmentation pattern for the augmented 1662 Da hemoglycin lattice molecule in the case of no Gly_OH_ residues. The primary break is symmetrical (red dashed line).

The 1494 Da “core” molecule of hemoglycin^3,6,8^ can be observed directly in mass spectrometry from purified samples^8^ but in the campaign that first revealed its existence^3^ the two dominant species beyond the core molecule contained one additional FeO unit (to yield *m*/*z* 1567) or two FeO units (to yield *m*/*z* 1639). It was proposed in ref. 5 that a three-dimensional lattice of the diamond 2H structure could have formed, and this structure was further confirmed in X-ray diffraction.^8^ This structure has tetrahedral symmetry at all vertices, leading to the belief^5^ that Si atoms with tetrahedral bond symmetry occupied these locations. With Si vertex atoms and additional oxygen atoms the (un-hydroxylated) 1638 Da molecule^3^ has augmented mass 1662 Da as shown in Fig. 8 and Table 3.

**Fig. 9:**
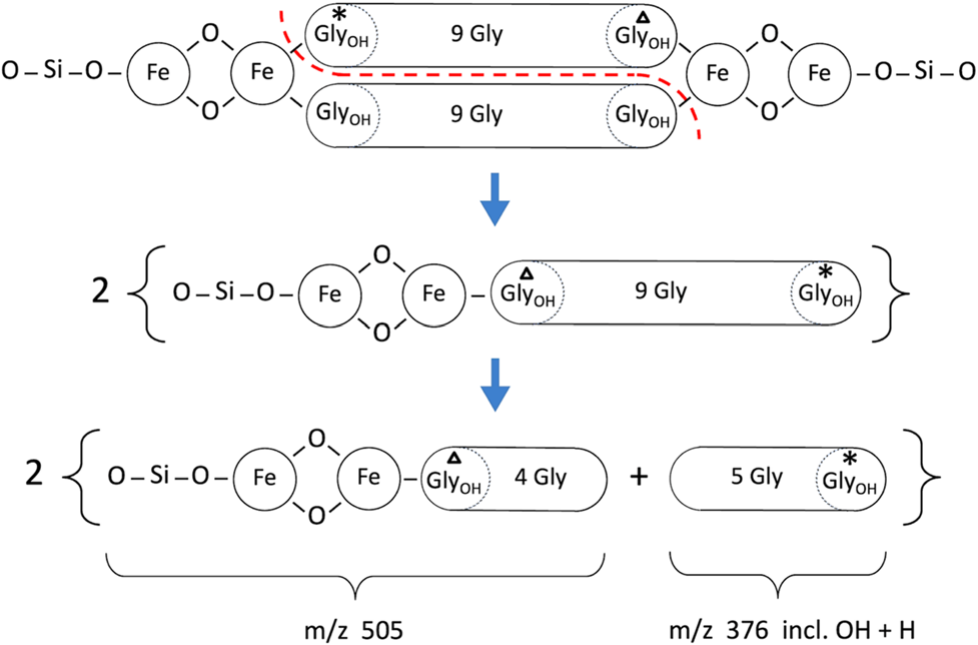
Fragmentation pattern for the augmented 1662 Da hemoglycin lattice molecule in the case of labelled Gly_OH_ residues that can be followed *via* identifying symbols. The primary break is a symmetrical splitting of the molecule (red dashed line).

Fragmentation data for the 1638 Da hemoglycin primary molecule augmented to 1662 Da for cases with or without hydroxyglycine (Gly_OH_), illustrated in Fig. 8 and 9^a^

**Table:** 

*m*/*z*	Molecule	CHCA_1	CHCA_2	SA_1	SA_2
1662	22Gly(Fe_2_SiO_4_)_2_	0	0	0	0

a (*) last row, *m*/*z* 206 for CHCA and *m*/*z* 204 for SA.

**Table:** 

*m*/*z*	Fragment	Counts			
		CHCA_1	CHCA_2	SA_1	SA_2
489	5GlyFe_2_SiO_4_	254 000	74 900	17 400	6000
505	4GlyGly_OH_Fe_2_SiO_4_	280 000	60 500	9400	5000
360	6Gly + OH + H	198 500	42 800	42 700	21 000
376	5GlyGly_OH_ + OH + H	161 300	24 200	53 500	29 800
190	3Gly + OH + 2H	51 500	130 000	20 600	8600
206*	2GlyGly_OH_ + OH + 2H	30 000	63 000	22 000	13 600

The principal fragments of the inferred *m*/*z* 1662 species, with either zero or one hydroxylation, are listed in Table 3. The signals were much lower with matrix SA and generally not at sufficient S/N to obtain definite Fe readings as found for example with CHCA in the 489 and 505 *m*/*z* species listed in Table 3.

**Fig. 10:**
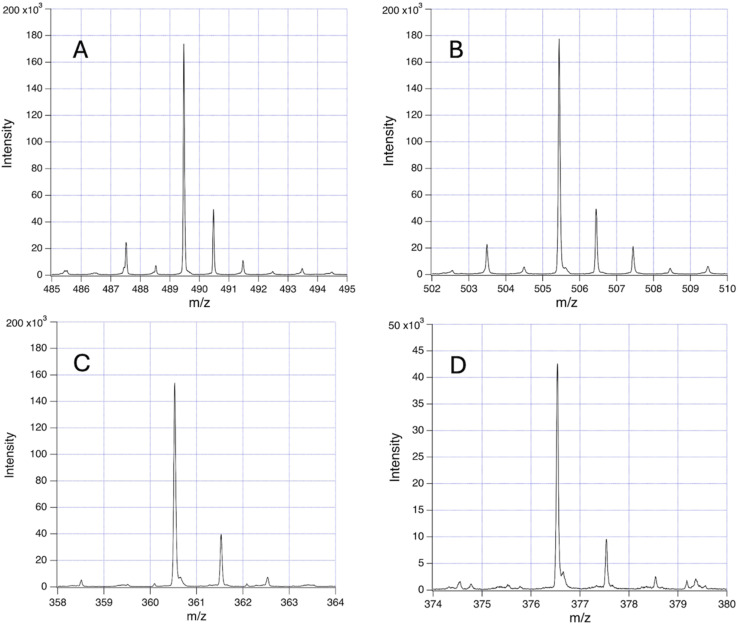
Illustrations of the isotopologue distributions for the fragments in Fig. 8 and 9: (A) 489 *m*/*z*; (B) 505 *m*/*z*; (C) 360 *m*/*z*, all with CHCA, and (D) 376 *m*/*z* with SA.

In Fig. 8 and 9 the primary break is again symmetrical (red dashed line), however different products are produced according to the number and disposition of Gly_OH_ residues that can be followed *via* the identifying symbols. As important as finding the identity of a given fragment is verifying the absence of certain related fragments. For example, in determining the structure of *m*/*z* 489 and its single hydroxylation *m*/*z* 505, it was seen that *m*/*z* 521 was absent, indicating that two hydroxylations were not present at a significant level in any one beta strand of this primary molecule. Fig. 10 presents a sample of the high S/N mass spectrometry traces that allowed Fe determinations *via* isotopologue analysis for the fragments in Fig. 8 and 9.

#### Observation of a “1662” *m*/*z* peak complex

**Fig. 11:**
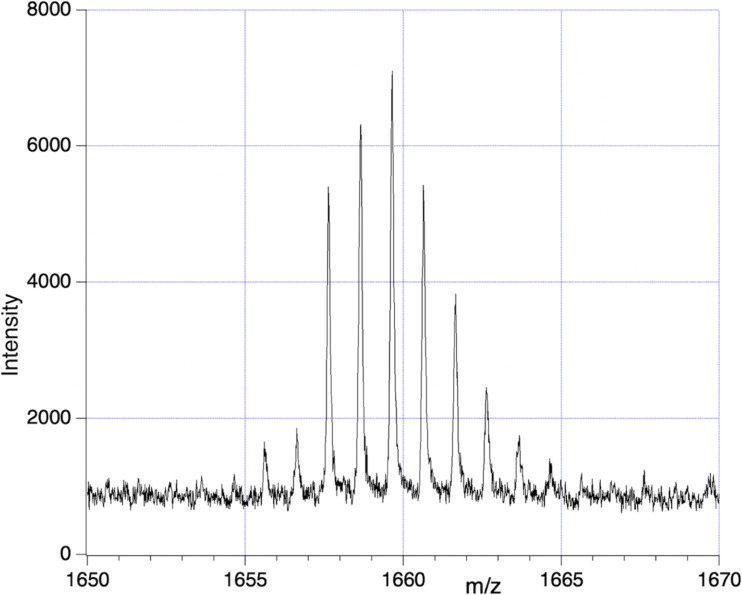
A complex molecular set observed in sample SF3 at approx. 1660 *m*/*z*.

Having traced in sample SF9 the fragment spectrum back to a 1662 Da entity a corresponding peak complex was found in sample SF3, although only in the SA matrix cases. It stood alone as the highest peak complex for *m*/*z* exceeding 1190. This complex, shown in Fig. 11, is at poor S/N but appears to have 3 or 4 Fe atoms, according to the relative height of the 1655/1656 isotopologues compared to the first large “mono-isotopic” peak at 1657 *m*/*z*. According to the fragment reconstruction in Fig. 8 and Table 2 there was predicted a species Gly_22_Fe_4_Si_2_O_8_ (*m*/*z* = 1662). It appears that in MALDI up to 5 protons can be missing from this complex molecule, the charge being compensated by Fe^+^ states. Apart from its low S/N, it appears that a variable number of proton adducts could be present, rendering isotope analysis impossible in this case either for Fe content or global ^2^H.

### Discussion of MALDI fragmentation results

The Fe content of fragments could be found by isotope analysis (Section S1†) for all of the species listed in Tables 2 and 3, with the exception of the 1200 series for which S/N was inadequate. Two consistent fragmentation sequences (Fig. 7–9) could be constructed that preserved original Fe configurations such as FeO_2_ and Fe_2_SiO_4_ and also followed the previously observed “un-zipping” fragmentation mechanism of the 1494 Da hemoglycin “core” molecule into *m*/*z* 730 moieties.^3^ The “1200 *m*/*z*” fragment sequence originated in a reduced version of the core molecule with 9 glycine residues per chain as opposed to 11. Quantum chemical modeling^6^ has shown that odd numbers of residues in the chain are more stable than even, due to the need to present converging free bonds toward the terminal Fe atoms. Only 11-residue chains have been observed in our prior work. The core chains in the present work are 9-residue (for 1200 *m*/*z*) and 11-residue (for 1662 *m*/*z*). The second fragment sequence derives from a 1662 Da core entity that was a silicon adduct of the 1638 Da molecule described in ^3^, itself based upon the “core” 22 residue hemoglycin core molecule but carrying four Fe atoms as opposed to two. The intensities of *m*/*z* peaks were very approximately five times smaller for the 1200 *m*/*z* set compared to the 1662 *m*/*z* set.

In each fragmentation sequence there were (+16) mass increments that are interpreted as varying degrees of hydroxylation of glycine. This was strikingly evident in the “1200” series (Fig. 6) where up to four hydroxylations were observed (Table 2). Prior mass spectrometry^3^ of meteoritic samples had shown that four hydroxyglycine residues was the standard, and the observation of a chiral 480 nm absorption^6^ pinned down the position of at least one hydroxyglycine residue as being adjacent to the terminal Fe atom. Possibly because the sea foam chemical environment is “active”, in water solution and exposed to visible/ultraviolet light, the proposed cycling mechanism associated with water-splitting^8^ is being observed. In this mechanism each glycine residue adjacent to Fe cycles between plain glycine and hydroxyglycine, which could give a reduced average “occupancy” compatible with the present observations. At face value, for the “1200” series, the average number of hydroxylations is 1.0 in the CHCA spectrum, and 0.9 in the SA spectrum of Fig. 6. The rough equality of the 489 *m*/*z* and 505 *m*/*z* fragment counts in Table 2 would suggest an average of 0.5 hydroxylations across the two main fragments in that sequence, summing to 1 hydroxylation in the complete 1662 species that is the whole molecule counterpart of the 1200 species, in qualitative agreement.

### Isotope analysis in MALDI

In the present paper, to give the isotopic analysis general utility we perform a “global” analysis as if ^2^H were the only enriched isotope (Method in Section S1†) (Table 4). If information subsequently becomes available either *via* a specific ^2^H or ^15^N measurement on a completely pure molecular sample then, under certain assumptions, the complementary enrichment of hydrogen or nitrogen can be estimated.

**Table:** Isotope measurements on main fragment species. “Global” enrichment represented by ^2^H per mil (‰) enrichment from fitted isotopologue intensities

Run	*m*/*z*	Formula	Enrichment (‰)
SF9 CHCA-2	360	6Gly + OH + H	20 000
SF9 (SA-1 + SA-2)	360	6Gly + OH + H	16 500
SF9 CHCA-1	376	5GlyGly_OH_ + OH + H	22 500
SF9 SA-1	376	5GlyGly_OH_ + OH + H	20 000
SF9 CHCA-1	489	5GlyFe_2_SiO_4_	20 000
SF9 CHCA-1	505	4GlyGly_OH_Fe_2_SiO_4_	21 000
SF9 CHCA-1	619	9GlyFeO_2_ + OH + H	15 000
SF9 CHCA-2	619	9GlyFeO_2_ + OH + H	17 500
SF9 CHCA-1	635	8GlyGly_OH_FeO_2_ + OH + H	12 500
		*n* = 9, ave. = 18 300, *σ* = 3000	

The enrichment of ^2^H relative to H is defined by the “per mil” *δ* (‰) measure:
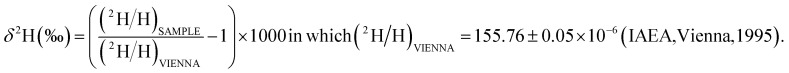


Controls for this isotope analysis were obtained from known matrix cluster peaks,^21^ giving a baseline of 700 ± 1700 (‰) enrichment for these terrestrial compounds (Section S1†).

## Discussion

This research suggests that the underlying structure of sea foam involves the polymer hemoglycin. The polymer could first form in the Universe once its constituent elements existed 13 billion years ago.^5,9^ The earliest references to sea foam (“aphros” = foam) occur in the Greek myth of the heavenly origin of Aphrodite^12^ without the then support of mass spectrometers, accurate telescopes and X-ray diffraction from synchrotrons. That relatively clean sea foam collected from two Rhode Island beaches contains the space polymer hemoglycin is deduced from the presence of vesicles after Folch extraction and from MALDI mass spectrometry. Vesicles present at the Folch extraction “interphase” layer are a key visual indicator that hemoglycin is in a sample, be it from meteorites,^3^ stromatolites^8^ or, in the present case, sea foam. The polymer of glycine and iron adopts a position in the two-phase Folch extraction interphase relative to its density and hydrophobic/hydrophilic structure. It is less dense than chloroform, residing at the top of the hydrophobic chloroform layer but remaining below the less dense hydrophilic methanol–water layer. Sand on the shoreline near to where foam landed did not contain hemoglycin and samples heated to 500C had the hemoglycin destroyed. The sea foam samples analyzed thus had properties that indicated they contained a molecule sensitive to heat and were not from a sand source at the shore-line. Space polymer in 100 micron scale in-fall particles potentially remains at the ocean surface and together with surface layer surfactants is incorporated *via* turbulent wave action into a foam, extracts of which retain the elevated heavy isotope levels characteristic of meteoritic or cometary material.

Cosmic material reaches Earth's surface as meteorites and also, more steadily and in larger quantities, as cosmic dust. Dust incident at orbital height above Earth's atmosphere has been measured by space probe to arrive at a rate of 4 ± 2 × 10^4^ metric tons per annum^22^ with a size distribution peaking at 200 microns diameter. Particles entering the atmosphere are heated by ablation in some cases to melting point, producing spherules, and in other cases losing mass by evaporation (categorized as un-melted micro-meteorites, or uMM). Combined averaged-over-time cosmic dust deposition in clean snow at Dome C, Antarctica^1^ amounts to 5200 + 1500/−1200 tonnes per annum when extrapolated to the whole globe, with size distribution peaking at about 100 microns diameter. In 26 years of walks on the two RI beaches we have only noted sea foam in the Autumn and early winter months and indeed the samples of the present work were gathered between 24th Oct 2023 and 27th Feb. 2024 (Table 1, sea foam samples). An exception to this pattern was the current year of 2024 where several spring to summer storms occurred. Two sea foam samples collected in mid-August 2024 after very turbulent seas due to the remnants of Hurricane Debby, produced typical vesicles after Folch extraction (Table 1). In search of an explanation we note that, because of the fixed tilt of Earth's axis,^11^ the Northern hemisphere is approaching the “zodiacal cloud” of meteoroids at one equinox, and the Southern hemisphere at the complementary equinox. Many other variables may be at work, such as more storms to agitate the sea surface in winter, or different surface microlayer surfactant content when the sea is colder. Also, meteor shower debris could contribute at other times.

As to the composition of in-falling dust, the picture can be complex, involving five major categories according to radar orbital analysis, with the principal category, amounting to 68% of the total, being the helion-antihelion fluxes.^23^ Containing cometary material, often from Jupiter family comets, these pass Earth en-route to perihelion close to the sun, and re-pass on the outward-bound leg at slightly reduced intensity. This group has relatively short collisional lifetime and must be constantly replenished by new cometary material on timescales of the order of 10^4^ years in order to remain the dominant apparent source of meteoroids.^23^ The properties of ultra-carbonaceous Antarctic micro-meteorites (UCAMMs)^24^ lend support to a cometary origin, for at least a fraction of the in-fall, in respect to their high N/C ratio.

Fine-grained uMM particles and UCAMMs have the highest content of “unaltered” carbon, within surviving organic molecules, and this may amount to 50 tonnes per annum when extrapolated to the globe,^1^ although this number is uncertain. The composition of the UCAMM organic material^24^ differs from carbonaceous meteorites in having a very high N/C ratio. Also, it has a strong infrared absorption in the region of 1600–1700 cm^−1^, a range corresponding to the amide I absorption of anti-parallel beta sheets of polymer amide.^9^ In the^24^ data there is also a prominent N–H vibrational mode absorption in the region of 3300 cm^−1^ that has been observed in FTIR of a meteoritic sample containing hemoglycin.^8,9^ While not providing conclusive proof that meteoroid infall is responsible for hemoglycin in sea foam, these facts are supportive.

As to the fate of in-falling 100 micron meteoroid particles of density about 2, it seems possible for many to be attracted into the sea surface layer of molecules known as the surface-microlayer (SML)^10^ which can be 50 microns thick and contains a wide range of organics, most resulting from the decay of organisms, as well as protein contributions by vegetation such as kelp.^16,17^ First contact of meteoroid dust with sea water will cause alkali metals to react, buoying the particle by gas release, and potentially causing organics to leach into the SML. Spherules, on average more massive and having smoothed surfaces, are more likely to sink to the sea floor. Infrared absorption of sea foam samples^15^ yields dominant amide I bands in the 1620–1700 cm^−1^ region, consistent with the presence of hemoglycin^9^ or other proteins with an anti-parallel beta sheet structure. Present-day stromatolite ooids contain hemoglycin as evidenced by X-ray induced fluorescence, X-ray scattering patterns and infrared absorption,^8^ thus confirming the availability of in-fall hemoglycin on a current basis in shallow, clear saline waters.

Isotope enrichment is observed in hemoglycin molecules identified by mass spectrometry^3,8^ and the same isotope analysis is here applied to the identified polymer amide molecules in Folch extracts of sea foam (details in Section S1†). Two heavy isotopes are substantially raised in cometary and primitive solar system material, namely ^15^N and ^2^H.^4^ In polymer amide from the Acfer 086 meteorite the ^15^N enrichment was 1015 (‰) *via* secondary ion mass spectrometry (SIMS),^4^ in the same category as cometary ^15^N enrichments.^25^^2^H enrichments of identified hemoglycin molecules are substantial, in the range 25 000 to 50 000 (‰), the latter number obtained from the Orgueil meteorite.^8^ If, as we believe, the sea foam hemoglycin is in-fall material, its original peptide backbone intact, then it still carries its original ^15^N enrichment trapped within in its repeating –CCN– backbone. On the other hand, the ^2^H atoms of hemoglycin slowly exchange with ^1^H of sea water. A distinction is made between the relatively rapid exchange of hydrogen in H–N bonds, and hydrogen in H–C bonds involving the alpha carbon of the peptide backbone. Overall, the rate of D–H exchange depends upon solution pH, and peptide conformation. For example,^26^ found a variable percentage of hard-to-exchange amide hydrogens (HEAHs) that increased to as much as 60% in insulin, attributed to a high content of strongly hydrogen-bonded secondary structures. Polyglutamic acid exchange at pD 3.5 did not reach completion in 168 hours, whereas N-deuterated proteins exchange relatively rapidly.^27^ In a simulation of the alkaline environment for meteoritic extraction^28^ measured a D–H exchange rate in dicarboxylic acid C–H bonds of (1.3–8.4) × 10^−4^ h^−1^ in solution at 100C. In summary, intact hemoglycin molecules are expected to retain their backbone ^15^N content, but to gradually lose their D content while afloat in the sea surface layer. Our data provides a “global” enrichment of 18 300 ± 3000 (‰) that includes ^15^N and ^2^H, within which there is likely to be the cometary ^15^N of 1040 ± 90 (‰).^25^ The hemoglycin molecule with H/N = 3 has a ^2^H decrease of 7.73 × (increase of ^15^N) (‰) (Section S1†), so to assume cometary ^15^N in the backbone leads to an estimated decrease of 8040 (‰) to global enrichment, giving 10 300 ± 4000 (‰) as a conditional estimate for ^2^H residual enrichment in sea foam hemoglycin. The removal of most of the expected 25 000–50 000 (‰) ^2^H enrichment for cometary hemoglycin (*via*,^3^ and Orgueil data^8^), thus implies that most of the deuteration in original cosmic dust may have been exchanged for seawater ^1^H by the time samples were collected.

Hemoglycin is present in sea foam, but does it contribute to the foam structure, or is it merely present as one of many organic components? The ready formation of vesicles by hemoglycin, mentioned above, could allow it to contribute to the structure of the very thin walls throughout sea foam. In prior work^3^ and an unpublished observation of X-ray scattering (Section S2†) we have found that hemoglycin rods can form a planar hexagonal lattice with side length of about 4 nm, the length of a 1494 Da core hemoglycin unit. Such an effectively two-dimensional lattice can, with small admixtures of pentagons or hexagons, cover arbitrary curved surfaces. In the Section S2† data, a crystal of hemoglycin derived from the Sutter's Mill meteorite^6^ contained a region that scattered x-rays into a simple set of six directions, the pattern persisting over a large range of incident angles, leading to the identification of scattering by a lenticular form, or collapsed vesicle, with multiple layers of this hexagonal lattice covering its surface. It is therefore possible to imagine that hemoglycin would be able to reinforce surfactant layers, allowing them to be very stable even as water drained from them, creating the very light and rugged foam that we observe. Finally, in regard to the propensity for sea foam to detach and fly in the wind, we have identified a possible water-splitting reaction cycle in which the hydroxyglycine next to a terminal iron atom can in two stages^8^ effect the overall reaction 2H_2_O + 2*hv* → H_2_ + H_2_O_2_, partially filling sea foam cavities with hydrogen.

## Methods

### Collection of sea foam samples

Sea foam was collected from 2 locations:

(1) Samples SF1-21 except sample SF17. Warrens Point Beach, Little Compton, RI 02837.

GPS: Latitude: 41.481924|Longitude: −71.144582.

(2) Sample SF17. Lloyds Beach, Little Compton RI 02837.

GPS: Latitude: 41.461357|Longitude: −71.196024.

The time of collection was determined by charts from US Harbors [https://www.ush.com/] on a daily basis from December 2023 to February 2024. Clean foam was obtained when a minimum low tide mark had been reached and the tide was incoming by 1–2 hours.

Sea foam is very stable and was collected in a wide necked glass container by scooping the foam from the sea directly into the container. It needs to be noted that once a small amount of foam is detached from a mass of foam floating on the sea or on the beach, it tends to become rapidly airborne, we believe because it contains a very low-density gas, probably hydrogen. Catching the foam in the container required positioning the container to use the wind direction to ensure the foam entered the container. It was then transported to the laboratory, chloroform added to dissolve, and poured into a glass V-vial. Methanol and some water were added, the whole V-vial gently vortexed to produce foam vesicles at the interphase of the chloroform : methanol : water (3.3 : 2 : 1) Folch extraction used previously for meteoritic samples^2^ [Fig. 2]. Fig. 3 shows a small amount of sea foam that was very clean, apparently containing only salts and hemoglobin. Fig. 4 shows collection and handling of a sample with a more dense amount of hemoglycin plus other secondary chemicals. Fig. 5 shows a very large amount of foam on the Lloyds beach location where the foam stretches hundreds of feet out to sea. This sample had many secondary substances present like sand and plant matter from the turbulent sea and represents a sample not analyzed because of contamination.

### MALDI mass spectrometry of sea foam samples

Intact stable vesicles from the Folch interphase layer were aspirated from the Sample SF3 V-vial (Fig. 2) into duplicate Eppendorf tubes containing the CHCA (α-cyano-4-hydroxycinnamic acid) or SA (sinapinic acid) matrix mixes described below. For Sample SF9 (Fig. 4) 200 μL of the yellow interphase layer were pipetted into duplicate Eppendorf tubes containing either CHA or SA matrix mix.

Mass spectrometry was performed on a Bruker Ultraflextreme MALDI-TOF/TOF instrument in the positive ion, reflectron mode. We used CHCA and SA matrices. Both were at 10 mg mL^−1^ in 50% acetonitrile in water, 0.1% trifluoroacetic acid in water. Our resolution was of the order of 10 000 and we looked in the range *m*/*z* = 0–5000, finding most peaks from 20–2000. A sample volume of 2 μL was mixed with a matrix volume of 2 μL, vortexed and left for one hour at room temperature. This one hour wait before pipetting 1 μL quantities onto the MALDI plate is essential to partly solubilize hemoglycin in the matrix solvents.

### X-ray structural analysis (Section S2†)

On Diamond beamline 124 diffraction data were recorded from the Sutter's Mill SM2 crystal at 1.000 A, 0.1° oscillation range, using a Pilatus3 S 6M detector.

## Data availability

The data that support the findings of this study are available from the corresponding author upon reasonable request and at the Harvard Dataverse repository *via* URL: https://doi.org/10.7910/DVN/A00GMD.

## Conflicts of interest

The authors report no conflict of interest.

## Supplementary Material

RA-014-D4RA06881E-s001
