# Supplementary material for: Sea foam contains hemoglycin from cosmic dust
Source: RSC Adv. 2024 Nov 20;14(50):36919–29. doi: 10.1039/d4ra06881e (PMC11576944; doi:10.1039/d4ra06881e)
Supplement: RA-014-D4RA06881E-s001 [file RA-014-D4RA06881E-s001.pdf]

Supplementary Information (SI) for RSC Advances.
This journal is © The Royal Society of Chemistry 2024

S Section: Sea Foam contains Hemoglycin from Cosmic Dust.

Julie E. M. McGeoch[1]and Malcolm W. McGeoch[2]

[1]High Energy Physics DIV, Smithsonian Astrophysical Observatory Center for Astrophysics | Harvard & Smithsonian, 60 Garden Str, MS 70, Cambridge MA 02138, USA
[2] PLEX Corporation, 275 Martine Str, Suite 100, Fall River, MA 02723, USA

S1 Isotope analysis

Molecular isotope analysis

In our prior work with meteoritic extracts [1, 2], or space-derived materials such as the hemoglycin lattice within stromatolites [2], the isotopologues around a "mono-isotopic" mass spectral peak have intensities that relate to the isotopic composition of each of the atoms in the molecule. The significant heavy isotopic enrichment of extraterrestrial glycine polymers becomes plainly visible in the isotopologue distributions for m/z values as low as 400, while at higher m/z the (+1), (+2), etc. peaks often rise even above the (0) "mono-isotopic" peak. Provided the molecule's atomic composition is known from fragmentation (e.g. MS/MS) studies, and it is known which elements have enrichment, a measure of a specific isotopic enrichment can be derived via a straightforward simulation [1]. Discussion in [1, S section] points to the likelihood that ^{15}N and ^{2}H are the two principal highly enriched isotopes in polymer amide extra-terrestrial material. The enrichment of ^{15}N can be isolated via separate study of CN negative ions from the molecule using secondary ion mass spectrometry (SIMS). We have measured a ^{15}N enrichment of 1,015 ± 280 ($^{0}/_{00}$) in polymer amide within the Acfer 086 meteorite, in broad agreement with cometary estimates [3], and it is presumed that because N is an integral part of the –CCN- repeating polymer backbone, any intact polymer in-falling to Earth, such as found in the present study, will by force contain the original extra-terrestrial ^{15}N within its backbone. The variations in ^{13}C are expected to be relatively less consequential in this molecular type [1, 3].

In the present paper, to give the isotopic analysis general utility we perform a "global" analysis as if ^{2}H were the only enriched isotope. If information becomes available either via a specific ^{2}H or ^{15}N measurement on a completely pure molecular sample then, under certain assumptions, the complementary enrichment of hydrogen or nitrogen can be estimated.

The enrichment of ^{2}H relative to H is defined by the "per mil" δ ($^{0}/_{00}$) measure:

$$\delta^{2}H\ (^{0}/_{00}) = \left(\frac{\left(^{2}H/H\right)_{SAMPLE}}{\left(^{2}H/H\right)_{VIENNA}} - 1 \right) \times 1000$$ in which $(^{2}H/H)_{VIENNA} = 155.76 \pm 0.05 \times 10^{-6}$ (IAEA, Vienna, 1995).

Without separate knowledge of ^{15}N enhancement in hemoglycin we fitted the prominent isotope enrichments as if ^{2}H was the only contributor. When ^{15}N values come available the quoted "global" ^{2}H enrichments may be modified as follows where Δ represents the degree of change:

$\Delta(\delta^2 H) = -7.73\Delta(\delta^{15} N)$. This relationship holds for polymers of glycine and hydroxyglycine that have $N_H/N_N = 3$. It may be scaled for general ratios of N_H to N_N via the relationship

$\Delta(\delta^2 H) = -(R_N N_N / R_H N_H)\Delta(\delta^{15} N)$ using the R_N and R_H values listed below.

The following isotope ratios (IAEA, Vienna, 1995) are taken as terrestrial standards:

VSMOW water	$R_H = {}^2H/{}^1H = 155.76 \pm 0.05 \times 10^{-6}$
VSMOW water	$R_O = {}^{18}O/{}^{16}O = 2{,}005.20 \pm 0.45 \times 10^{-6}$
V-PDB	$R_C = {}^{13}C/{}^{12}C = 11{,}237.2 \times 10^{-6}$
Atmospheric Nitrogen	$R_N = {}^{15}N/{}^{14}N = 3{,}612 \pm 7 \times 10^{-6}$

Figure S1.1 shows a typical fitted "global" isotope enrichment, in ^2H equivalent. The curve is for an assumed 21,000 mil ($^0/_{00}$) ^2H enrichment with all other isotopes set at terrestrial (Vienna) level. The (-1) and (-2) components are at the level indicative of 2 Fe atoms in the m/z 505 entity. In the main text the possibility of a ^{15}N component to the global isotope enhancement is considered.

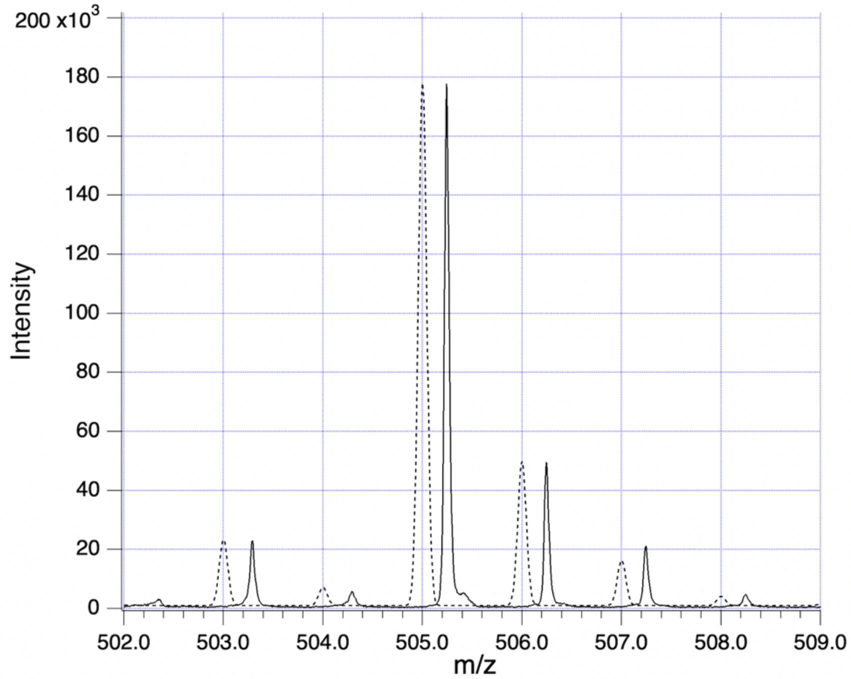

Figure S1.1. Isotope match of the m/z 505 fragment in sample run SF9-CHCA1, showing 2Fe via ^{54}Fe content at m/z 503. Data, solid line. Fitted curve, dashed line, normalized at peak and offset for clarity.

Global isotope enrichments for the main fragment species are listed in Table S1.1. There is an average global "^2H" enrichment of 18,300 ± 3,000 ($^0/_{00}$), which appears to indicate significant isotope enrichment <u>in the hemoglycin component</u> of sea foam (which is not to say that it would be measurable in sea foam as a whole, with its many additional organic components).

A convenient control for isotope levels in the m/z range of interest comes from MALDI matrix clusters that are well-characterized [4] and terrestrial to the accuracy required for the present work. We found high S/N matrix clusters in CHCA for three samples that had lower hemoglycin content and largely empty spectra apart from matrix peaks (SF3, SF8 and SF16). The isotope analysis was applied to these matrix clusters of known atomic contents with results shown in Table S1.2. Without any change to the Vienna set (above) the fits all indicated a small negative δ^2H except for one. Negative values were not included in the average because the enrichment becomes very nonlinear – for example, isotopically pure H would have δ^2H = -1,000 ($^0/_{00}$). Variation of $\delta^{13}C$ to -100 ($^0/_{00}$) is sufficient to fit most of the matrix cluster isotope patterns at otherwise terrestrial (Vienna) values. The positive global enhancements in sea foam hemoglycin (Table S1.1) are therefore statistically real, although their isotopic breakdown is not available from this approach.

Table S1.1 Isotope measurements on main fragment species. "Global" enrichment represented by ^2H per mil ($^0/_{00}$) enrichment from fitted isotopologue intensities.

run	m/z	Formula	Enrichment ($^0/_{00}$)
SF9 CHCA-2	360	6Gly + OH + H	20,000
SF9 (SA-1 + SA-2)	360	6Gly + OH + H	16,500
SF9 CHCA-1	376	$5GlyGly_{OH}$ + OH + H	22,500
SF9 SA-1	376	$5GlyGly_{OH}$ + OH + H	20,000
SF9 CHCA-1	489	$5GlyFe_2SiO_4$	20,000
SF9 CHCA-1	505	$4GlyGly_{OH}Fe_2SiO_4$	21,000
SF9 CHCA-1	619	$9GlyFeO_2$ + OH + H	15,000
SF9 CHCA-2	619	$9GlyFeO_2$ + OH + H	17,500
SF9 CHCA-1	635	$8GlyGly_{OH}FeO_2$ + OH + H	12,500
		n = 9 ave. = 18,300 σ = 3,000	

Table S1.2. Control isotope measurements on CHCA matrix species. "Global" enrichment represented by ^2H per mil ($^0/_{00}$) enrichment from fitted isotopologue intensities. M = CHCA = $C_{10}H_7NO_3$.

run	m/z	Formula	Enrichment ($^0/_{00}$)
SF16 CHCA-1	445	$M_2Na_3H_{-2}$	< 0
SF16 CHCA-2	445	$M_2Na_3H_{-2}$	< 0
SF16 CHCA-1	656	$M_3Na_4H_{-3}$	< 0
SF8 CHCA-2	656	$M_3Na_4H_{-3}$	< 0
SF3 CHCA-1	861	$M_4KNa_3H_{-3}$	< 0
SF16 CHCA-2	861	$M_4KNa_3H_{-3}$	5,000
SF3 CHCA-1	877	$M_4K_2Na_2H_{-3}$	< 0
		n = 7 ave. = 700 σ = 1,700	

S2. Meteoritic evidence for a hemoglycin hexagonal surface-covering mesh

S2.1 Introduction

In this section we discuss a possible structural role for hemoglycin in the stabilization of sea foam bubbles. Its first chemical identification [1] was in CV3 class carbonaceous meteorites, particularly Acfer 086. A crystalline extract via Folch solvent from Sutter's Mill meteorite showed the characteristic square mesh structure of hemoglycin via its x-ray scattering [5]. However, in one crystal location, it displayed an extremely simple hexagonal X-ray pattern that we identified as being from a vesicle trapped within the sheet crystal. The vesicle appears to consist of a surface-covering hexagonal mesh composed of triskelia, or three-legged subunits, in which each "leg" is based upon a hemoglycin core molecule. The ability of hemoglycin to form curved surface sheets may contribute to the formation and stabilization of sea foam, hence the following summary of this unpublished X-ray observation.

Solvent extracts from meteorites frequently display floating small globules with a dark surface layer that remain stable for months [1], referred to here as vesicles (Fig. S2.1). Previously, X-ray data from hemoglycin fibers has revealed that they have an extended square lattice with 1494Da connecting rods [6]. The X-ray scattering is dominated by iron atoms at the junctions of these rods. Apart from fibers, hemoglycin also crystallizes into sheets. A flat crystal from an extract of the Sutter's Mill meteorite (sample SM2) has exhibited the typical ladder of diffracted orders associated with this square lattice, extrapolating to a first order spacing of 48-49 Angstroms [5].

In an extended exploration of crystal SM2 the 50micron I24 beam of the Diamond Light Source (UK) was aimed at different positions, revealing an unusual diffraction pattern in the eighth data run. Although there was weak presence at this location of the square lattice diffraction ladder, a much stronger feature was seen for the first time, comprising an exact hexagonally disposed set of short diffraction arcs, spaced on a well-defined ring at 4.085 Angstroms. A frame containing this is shown in Figure S2.2.

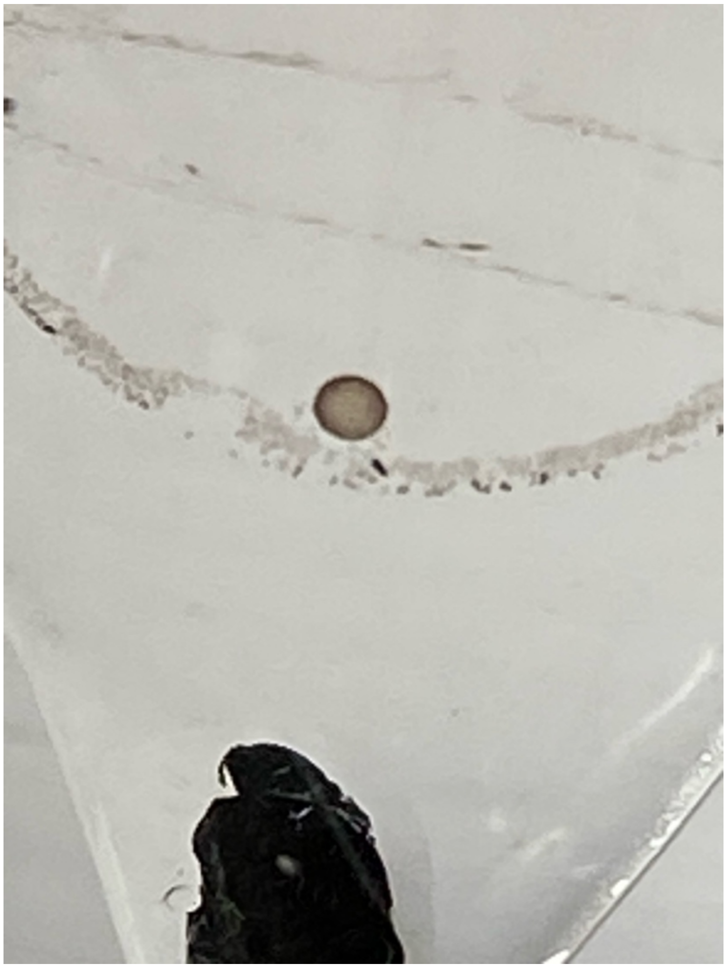

FIGURE S2.1. Sutter's Mill meteorite particle undergoing Folch extraction in a borosilicate glass V-vial. A spherical dark 533µm diameter vesicle of hemoglycin (image lit from the side) has formed at the interphase between the dense chloroform phase and the polar methanol/water phase. The dark object at the bottom of the vial is the particle of Sutter's Mill meteorite from which the polymer diffuses on solvation. The polymer vesicle resides at the interphase because it is less dense than chloroform but denser than methanol/water due to hydrophobic poly-glycine content.

This pattern persisted for about 600 images, (i.e. about 60°) at each of two 180° opposed locations within a 360° scan. Because it persisted over such a large range of incident angles the object had to have curvature. The full azimuthal distribution of the pattern ruled out the one-dimensional curvature of fiber diffraction, which shows a narrow line of orders [5, 6] leading out from the central beam stop. The possibility of diffraction from a two-dimensional region of curvature was therefore considered. This could not have involved a full sphere in view of the limited persistence across frames of the observed diffraction. However, there could have been a flattened lenticular form if a spherical vesicle had collapsed, and this would show diffraction over a more limited angle range, as illustrated in Figure S2.3.

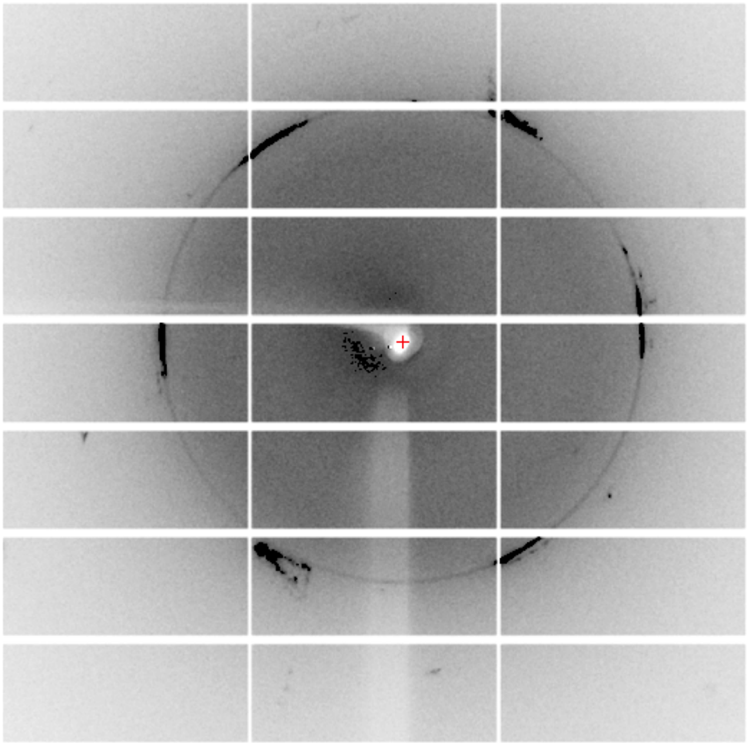

Figure S2.2 Frame 3100 of Sutter's Mill crystal SM2 at 1.000A, 0.1^{O} oscillation range. The thin circle represents 4.085A spacing.

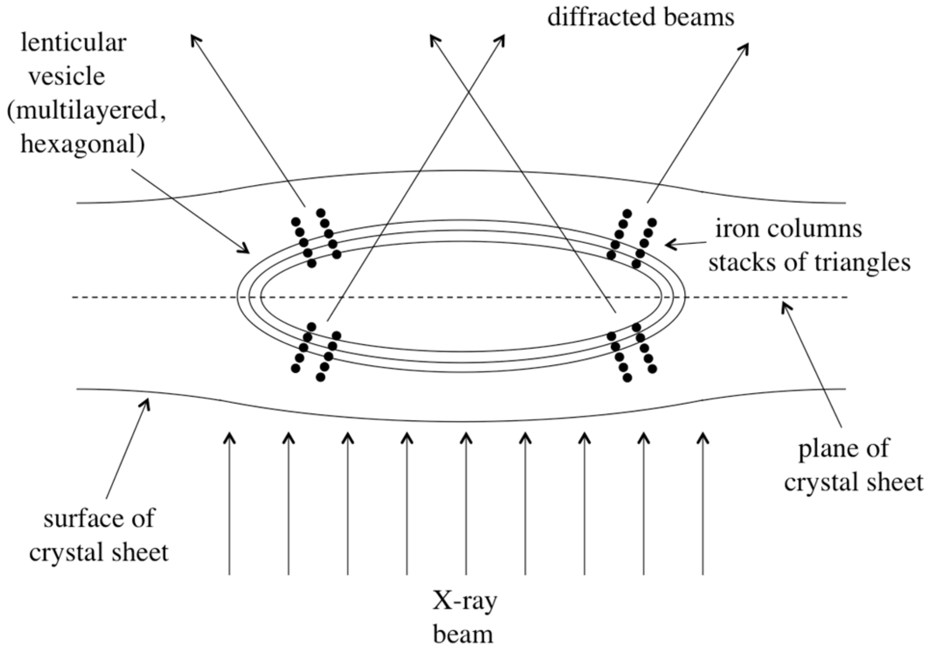

Figure S2.3. Edge view of a lenticular inclusion in a sheet crystal that could have originated as a spherical vesicle trapped between sheets of the crystal, then dried by evaporation.

In Figure S2.4 we show the lattice structure that appears to be responsible for the hexagonal pattern of Figure S2.2. This is based upon polymer rods of hemoglycin that are bonded in three-way junctions (also called triskelia) as proposed in [1], able to cover flat or curved areas in combinations of hexagons and pentagons. At each junction there are three iron atoms arranged in a triangle, represented by small dark circles in Figure S2.4 with dotted connections representing either Fe-O-O-Fe or Fe-O-Si-O-Fe. The second of these has been implicated by a mass spectrometry match in the m/z 4641Da entity [1]. However, the first of these, without Si, appears to match the present Sutter's Mill data, being in agreement with a new MMFF simulation that predicts 4.2A separation between Fe atoms in the Fe-O-O-Fe case in good agreement with the observed spacing of 4.085A.

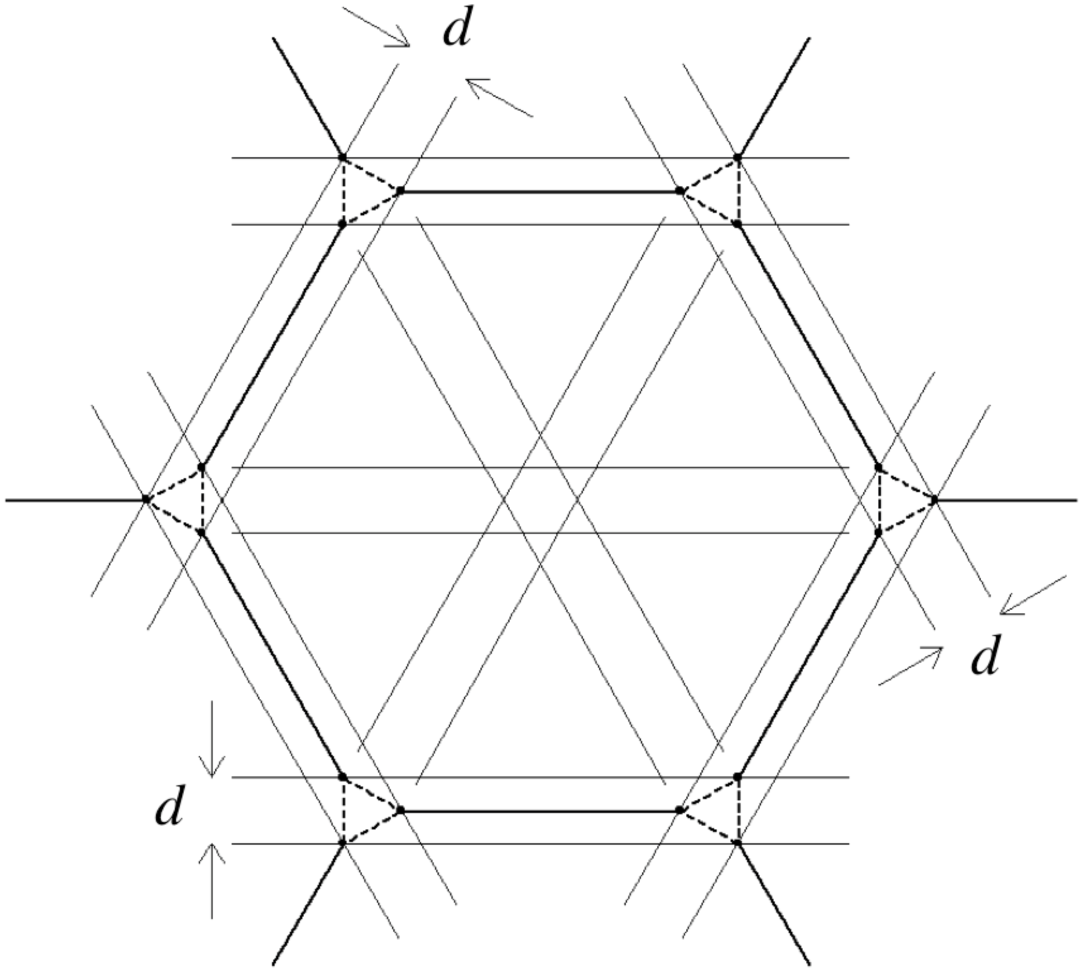

Figure S2.4. Diffracting planes of separation *d* in a hexagonal lattice with Fe atoms at triangular junctions. The junctions are shown at 2x scale for clarity.

In Figure S2.4 there are three orientations for scattering, each with the same inter-plane spacing *d*. This structure, when layered over a curved surface will create the opposed pairs of scattering resonances pictured in Figure S2.2, totaling six, as observed. Only a single spacing *d* is possible, not considering the full diagonal of the hexagon at a spacing of about 76 Angstrom that cannot be resolved because it lies above the beam stop limit of about 45A.

In Figure S2.5 we review the hemoglycin 1494Da "core" polymer, here pictured in its triskelion form with an inset showing the triangle of iron atoms at each triskelion junction. The existence of a triskelion had been proposed in [1] to explain the 4641Da entity previously seen via bonding of three of the newly derived 1494Da core units, together with mass spectrometry Na adducts. The version shown does not have Si in the junctions, to match the 4.085A Fe spacing seen here.

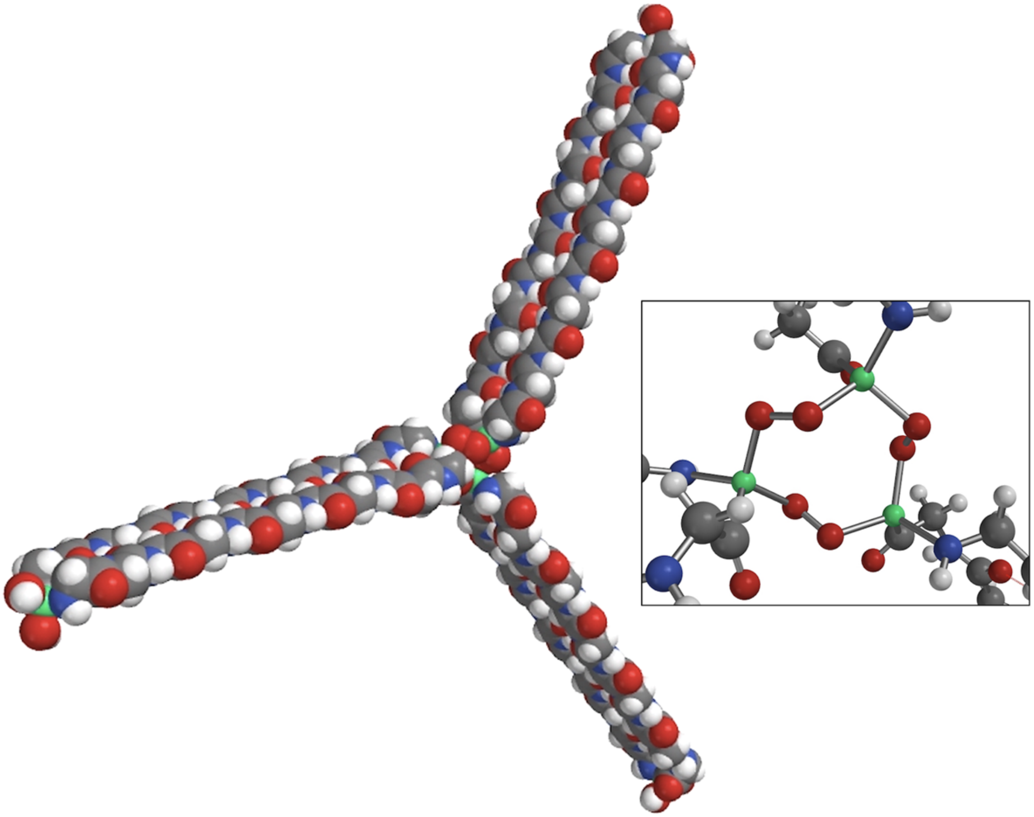

Figure S2.5. Space-filling representation of the triskelion component of the hexagonal lattice. Fe green, O red, N blue, C black, H white. Insert, the junction region, ball and spoke format.

We are led to propose that a hemoglycin vesicle of structure different to the bulk sheet crystal has been incorporated and partly flattened, creating a lenticular form as drawn viewed edge-on in Figure S2.3. Although composed of the 1494Da core unit, like the main crystal sheet, this vesicle would have been clad in a hexagonal mesh of triskelia.

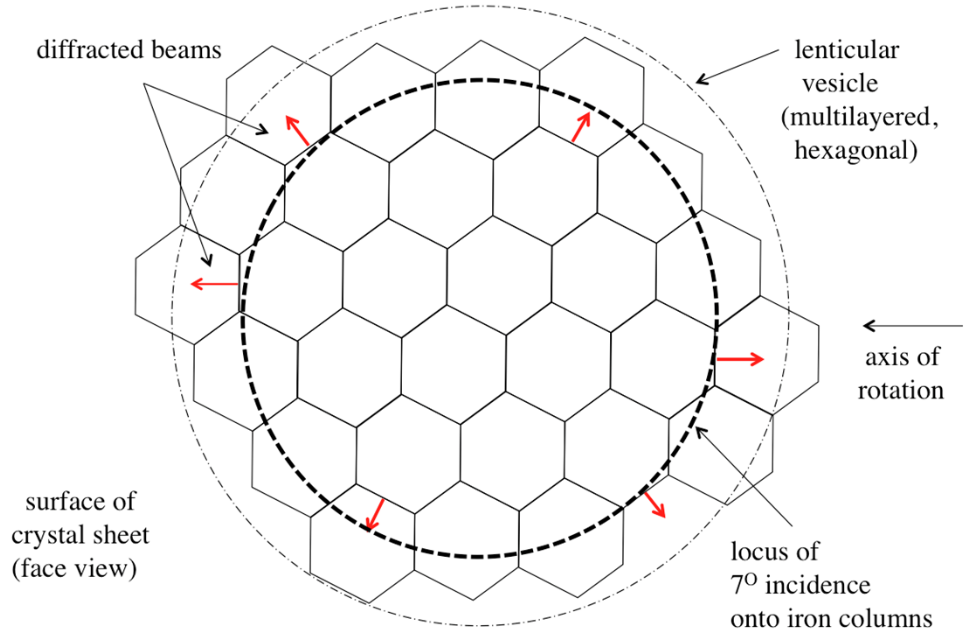

Figure S2.6. Face view of lenticular hexagonal-surfaced vesicle showing the locus where the 7° Bragg angle applies, and the six scattering directions. The X-ray beam is perpendicular to the page. Either face of the vesicle can contribute to the scattering.

Section S2. Acknowledgements
The authors thank Diamond Light Source UK for beamtime (proposal MX31420) and Robin Owen, Sofia Jaho and Sam Horrell for hemoglycin crystal X-ray data collection on beamline I24. The Sutter's Mill meteorite sample was kindly provided by Michael E. Zolensky of NASA.

References for S1 and S2

1. M. W. McGeoch, S. Dikler and J. E. M. McGeoch, Meteoritic Proteins with Glycine, Iron and Lithium", 2021, https://arxiv.org/abs/2102.10700 [physics.chem-ph]
2. J. E. M. McGeoch, A. J. Frommelt, R. Owen, G. Cinque, A. McClelland, D. Lageson and M. W. McGeoch, Fossil and present-day stromatolite ooids contain a meteoritic polymer of glycine and iron, *Int. J. Astrobiology*, 2024, **23** (e20), 1–21 doi:10.1017/S1473550424000168
3. M. W. McGeoch, T. Šamoril, D. Zapotok and J. E. M. McGeoch, Polymer amide as a carrier of ^{15}N in Allende and Acfer 086 meteorites, 2018, https://arxiv.org/abs/1811.06578
4. W. A. Harris, D. J. Janecki and J. P. Reilly, The use of matrix clusters and trypsin autolysis fragments as mass calibrants in matrix-assisted laser desorption/ ionization time-of- flight mass spectrometry, *Rapid Commun. Mass Spectrom.*, 2002, **16**, 1714-1722 https://doi.org/10.1002/rcm.775
5. J. E. M. McGeoch and M. W. McGeoch, Chiral 480nm absorption in the hemoglycin space polymer: a possible link to replication, *Scientific Reports*, 2022, **12**, 16198 doi: 10.1038/s41598-022-21043-4
6. J. E. M. McGeoch and M. W. McGeoch, Structural Organization of Space Polymers, *Physics of Fluids* 2021, **33(**6) doi:10.1063/5.0054860.
